# Population Pharmacokinetics and Initial Dosage Optimization of Tacrolimus in Pediatric Hematopoietic Stem Cell Transplant Patients

**DOI:** 10.3389/fphar.2022.891648

**Published:** 2022-07-06

**Authors:** Xiao-Lin Liu, Yan-Ping Guan, Ying Wang, Ke Huang, Fu-Lin Jiang, Jian Wang, Qi-Hong Yu, Kai-Feng Qiu, Min Huang, Jun-Yan Wu, Dun-Hua Zhou, Guo-Ping Zhong, Xiao-Xia Yu

**Affiliations:** ^1^ Department of Pharmacy, Sun Yat-sen Memorial Hospital, Sun Yat-sen University, Guangzhou, China; ^2^ Institute of Clinical Pharmacology, School of Pharmaceutical Sciences, Sun Yat-sen University, Guangzhou, China; ^3^ Department of Paediatrics, Sun Yat-sen Memorial Hospital, Sun Yat-sen University, Guangzhou, China

**Keywords:** tacrolimus, pediatric HSCT patients, population pharmacokinetics, Bayesian estimation, dosage simulation

## Abstract

**Background:** There is a substantial lack of tacrolimus pharmacokinetic information in pediatric hematopoietic stem cell transplant (HSCT) patients. This study aimed to develop population pharmacokinetics (PopPK) of tacrolimus in pediatric HSCT patients and to devise model-guided dosage regimens.

**Methods:** A retrospective analysis was performed on 86 pediatric HSCT patients who received tacrolimus intravenously or orally. A total of 578 tacrolimus trough concentrations (C_0_) were available for pharmacokinetic analysis using a non-linear mixed-effects modeling method. Demographic and clinical data were included and assessed as covariates *via* the stepwise method. Bayesian estimators were used to devise pediatric dosage regimens that targeted C_0_ of 5–15 ng mL^−1^.

**Results:** A one-compartment model with first-order absorption adequately described the tacrolimus pharmacokinetics. Clearance (CL), volume of distribution (V), and typical bioavailability (F) in this study were estimated to be 2.42 L h^−1^ (10.84%), 79.6 L (16.51%), and 19% (13.01%), respectively. Body weight, hematocrit, post-transplantation days, and caspofungin and azoles concomitant therapy were considered significant covariates for tacrolimus CL. Hematocrit had a significant impact on the V of tacrolimus. In the subgroup cohort of children (*n* = 24) with *CYP3A5* genotype, the clearance was 1.38-fold higher in CYP3A5 expressers than in non-expressers. Simulation indicated that the initial dosage optimation of tacrolimus for intravenous and oral administration was recommended as 0.025 and 0.1 mg kg^−1^ d^−1^ (q12h), respectively.

**Conclusion:** A PopPK model for tacrolimus in pediatric HSCT patients was developed, showing good predictive performance. Model-devised dosage regimens with trough tacrolimus concentrations provide a practical strategy for achieving the therapeutic range.

## Introduction

Tacrolimus (FK506), an immunosuppressive drug, has been widely used in hematopoietic stem cell transplant recipients to prevent chronic and acute graft-versus-host disease (GVHD). Evidences from clinical trials have shown that although calcineurin inhibitors with short-term methotrexate constitute GVHD prophylaxis in HSCT, standard tacrolimus-containing regimens have more favorable outcomes than that with cyclosporine A (Nash et al., 2000; Hiraoka et al., 2001; Oshima et al., 2010). Adequate post-transplantation immunosuppressive therapy is essential to prevent GVHD, whereas excessive immunosuppression may lead to serious infections, toxicities, and even increased risk of further malignancy.

Given the majority of literatures related to tacrolimus therapy in HSCT patients, adequate exposure to ensure graft outcomes but with minimum side effects should be guided by therapeutic drug monitoring (TDM) based on the C_0_ concentration, and the therapeutic range is confirmed as 5–15 ng mL^−1^. Tacrolimus therapy is challenging due to its significant inter-/intra-patient variation in pharmacokinetic variability, narrow therapeutic index, and poor oral bioavailability. These factors cause that even minor variations in its exposure may result in reduced immunosuppression or drug toxicity with potentially serious consequences. For example, the neurotoxic and nephrotoxic adverse effects of tacrolimus are severe and occur frequently, where associations to tacrolimus trough concentrations over 20 ng mL^−1^ have been documented.

Pharmacokinetic variabilities in tacrolimus are thought to be particularly significant in clinical application. It is mainly metabolized by cytochrome P450 3A4 and 3A5 (CYP3A4/5), and the clinical pharmacogenetics implementation consortium (CPIC) guideline has been issued to give recommendations for initial tacrolimus dose according to CYP genotypes (Birdwell et al., 2015). In addition, considerable between-subject variability can be attributed to various intrinsic factors, including patient age, race, hematocrit, liver function, albumin levels, and P-glycoprotein expression (Staatz and Tett,^2004^; Brooks et al., 2016). Beyond intrinsically high pharmacokinetic variability, tacrolimus is also susceptible to concomitant medication, donor factors, and food administration. Notorious inhibitors of CYP3A4/5 (such as azole antifungals) reportedly influence the metabolism if used in combination with tacrolimus. Antifungals, used frequently in HSCT patients, make a complicated impact on achieving the target C_0_ of tacrolimus. Therefore, individualized tacrolimus dosage, but not empiric adjustment, is imperative to obtain the desired trough concentration in HSCT patients, particularly in the pediatric HSCT population whose pharmacokinetics of drugs are quite different from adult patients.

To minimize side effects and instruct individualized tacrolimus dosing, it is necessary to gather pharmacokinetic information on tacrolimus in pediatric HSCT patients. Population pharmacokinetic model is an effective tool to investigate and quantify intra- and inter-individual variation by sparse sampling, especially in special populations that have difficulties in blood sampling, such as pediatrics. The population pharmacokinetics of tacrolimus in HSCT recipients have been studied in adults and children (Jacobson et al., 2001; Wallin et al., 2009; Xue et al., 2009; Wang et al., 2020; Brooks et al., 2021; Yoshida et al., 2021; Zhou et al., 2021). However, the evidences are somewhat limited by the relatively small sample size, lack of significant covariates (such as CYP3A5*1 polymorphism), and indeterminate drug combination. Otherwise, tacrolimus dosage schedules for the pediatric HSCT recipients are often prescribed as continuous intravenous (IV) administration initially, followed by oral therapy. With alternation of the administration route continually, the risk of developing tacrolimus-related toxicities would increase to a certain extent. Thus, according to the target concentrations promptly to the changing needs of the dosage adjustment, avoiding excessive or lower tacrolimus concentrations. Low tacrolimus concentrations are associated with an increased risk of acute rejection events, while higher amounts seem to be related to toxicities. Lack of information on the pharmacokinetic profiles of tacrolimus and on factors that cause individual variability makes it difficult to easily individualize tacrolimus dosage in pediatric HSCT patients.

Our study aimed to develop a PopPK model of tacrolimus in pediatric HSCT patients, describe the population pharmacokinetics of tacrolimus and evaluate clinical covariates that affect the pharmacokinetic parameters, and devise model-guided dosage regimens to assist with continuous IV and oral therapy.

## Materials and Methods

### Study Population

A total of 86 pediatric patients who underwent hematopoietic stem cell transplantation between January 2017 and December 2020 in Sun Yat-sen Memorial Hospital were included in the study. Patients aged 0–18 years, undergoing bone marrow, peripheral blood, or cord blood transplantation, were enrolled in the retrospective study. Each eligible patient received tacrolimus by continuous 24 h intravenous infusion and orally administration after the operation. The study was approved by the ethics committee of Sun Yat-sen Memorial Hospital (No. SYSEC-KY-KS-2020-163). Written informed consent was obtained from a parent or legal guardian.

### Data Collection

Whole-blood trough concentrations of tacrolimus were collected from therapeutic drug monitoring (TDM) records. Blood concentrations of tacrolimus were determined by using an enzyme multiplied immunoassay technique (EMIT) assay with the Viva-E^®^ System (Siemens, Germany).

Laboratory tests, demographic characteristics, and concomitant mediations were collected retrospectively from medical records, including gender, age, weight, diagnoses, transplant type, graft-versus-host disease (GVHD) grade, post-transplant days (PTD), white blood cells (WBC), red blood cells (RBC), hemoglobin (Hgb), platelets (PLT), hematocrit (HCT), urea (Ur), serum creatinine (SCr), total protein (TP), albumin (ALB), globulin (GLB), alanine transaminase (ALT), aspartate transaminase (AST), and total bilirubin (TBIL). Concomitant drugs included methotrexate, methylprednisolone sodium succinate, antifungal drugs (voriconazole, itraconazole, posaconazole, and caspofungin), antiviral drugs (acyclovir, valacyclovir, ganciclovir, and oseltamivir), and proton pump inhibitors (omeprazole and lansoprazole).

### Genotyping of *CYP3A5* Polymorphism

Ethylenediaminetetraacetic acid-anticoagulated whole blood (2 ml) was gathered from 24 hematopoietic stem cell transplant recipients (subpopulation). DNA was extracted from peripheral blood using the TIANamp blood DNA kit (Tiangen, China). CYP3A5*1 polymorphisms were genotyped by using the MassARRAY^®^ MALDI-TOF system. The primer sequences are presented in Supplementary Table S1. Deviations in allele frequencies from the Hardy–Weinberg equilibrium were tested using Pearson’s chi-square test.

### Model Development

The concentration-time data for tacrolimus was modeled by the first-order conditional estimation with interaction (FOCE-I) using a nonlinear mixed-effects method in Phoenix NLME™ (version 7.0; Certara L.P Pharsight, MO, United States). GraphPad Prism (version 8.2.1 Windows version, GraphPad Software, San Diego) was used for graphical analysis. The model was parameterized in terms of absorption rate constant (K_a_), clearance (CL), and volume of distribution (V). One-compartment models with or without lag time were evaluated to describe the tacrolimus concentration-time profile during the model development. Between-subject variability (BSV) was assessed for pharmacokinetic parameters using an exponential error model shown in the following equation (Eq. 1):

$$P_{j}=P\cdot \exp\left(\eta_{j}^{p}\right),$$ (1)

where P is the typical population value of the estimated parameters, P_j_ is the value of the *j*th individual, and η_j_^P^ represents the interindividual variability. *η*_j_ follows the normal distribution around 0 with the variance of ω^2^.

Additive, proportional, or mixed (additive and proportional) models were evaluated to describe the residual error.

### Covariate Analysis

All potential covariates were tested using a stepwise method with a forward-inclusion process and a backward-exclusion process. During the forward selection, a covariate was retained in the model if a significant (*p* < 0.05) decrease in the OFV (reduction>3.84) from the basic model was obtained. The importance of each variable was then re-evaluated by backward selection. An increase in the OFV of more than 10.83 (*p* < 0.001) was required for confirmation.

Improvement in goodness-of-fit plots and parameter estimation precision, reduction in between-patient variability and residual error, and stability of the parameter estimates were also taken into account to select the covariates.

Continuous covariates were tested by the power equation (Eq. 2):

$$P=P^{\prime }.{\left[\mathrm{Co}v_{\mathrm{con}}/\mathrm{median}\left(\mathrm{Co}v_{\mathrm{con}}\right)\right]}^{\mathrm{\theta cov}\_\mathrm{con}},$$ (2)

where P′ is the typical population value of the pharmacokinetic parameter, θ_cov_ is the estimated coefficient of the covariate, and the continuous covariate (Cov_con_) was normalized by its median value.

Categorical covariates were tested by the following equation (Eq. 3):

$$P=P^{\prime }\cdot \exp\left(\theta_{\mathrm{cov}\_\mathrm{cat}}\cdot \mathrm{Co}v_{\mathrm{cat}}\right),$$ (3)

where Cov_cat_ is set to 0 or 1 for categorical covariates.

To evaluate the effect of CYP3A5 genotypes on the CL, a subpopulation dataset (model 2) was developed based on the established PopPK model (model 1), incorporating genotype information. CYP3A5 polymorphisms were defined as binary covariates, whereas ‘1’ represented CYP3A5 *3/*3(CYP = 0) and* “2”* represented CYP3A5 *1/*1 or CYP3A5 *1/*3(CYP = 1). The use of azole antifungal drugs and proton pump inhibitors were merged into two variables: *“*CZ*”* and *“*PZ*”*, as the number of patients using a single drug was small. Post-transplantation day (PTD) was defined as a categorical covariate: PTD = 1 when post-transplantation day was less than 28 days, whilst PTD = 2 when post-transplantation day was more than 28 days (Wallin et al., 2009).

### Model Qualification

The goodness of fit was assessed graphically and numerically. Model diagnostic graphs included observed values versus population predictions and individual predictions; conditional weighted residuals (CWRES) versus population predicted values or elapsed time; CWRES and individual weighted residuals (IWRES) Quantile–Quantile Plot.

A nonparametric bootstrap with 1,000 times re-sampling was performed to evaluate the model performance and robustness. The validity of the model was evaluated by comparing the median estimates and their corresponding 95% confidence intervals from the bootstrap procedure with those estimated from the original dataset.

Prediction-corrected visual predictive check (pcVPC) was also used to assess the predictive performance of the final model. Simulation of 1,000 new replicates was conducted using the final model with estimated fixed- and random-effects model parameters. The concentration-time profiles were plotted for the 5th, 50th, and 95th percentiles (presenting the 90% prediction interval) of the simulated data and were overlaid with observed data.

### Simulation

The validated final model (model 1) with estimated fixed- and random-effects pharmacokinetic parameters was applied to simulate under different dosages of continuous 24 h intravenous infusion (0.01, 0.025, and 0.05 mg kg^−1^ day^−1^) or twice-daily oral administration (0.05, 0.1 and 0.2 mg kg^−1^ day^−1^, q12h). For each situation, 1,000 replications were performed. The median and 90% confidence intervals of C_0_ were generated. Different Hgb levels were set at 70, 90, 110, and 130 g L^−1^ according to the WHO Hemoglobin Concentrations for the Diagnosis of Anemia and Assessment of Severity (World Health Organization, 2011).

The subpopulation model (model 2) combined with the CYP3A5 genotype was used to simulate the C_0_ in children with different weights and CYP3A5 genotypes. For each medication scenario, 1,000 replications were performed. The median and 90% confidence interval were calculated. Based on the simulation results, the recommended doses for intravenous infusion and oral administration are given when the target therapeutic concentration is 5 ng mL^−1^ and 10 ng mL^−1^, respectively.

## Results

### Participants’ Characteristics

Data from 86 enrolled patients (median (range) age 5 (1–16), 53 boys and 33 girls) were analyzed (Table1). A total of 578 tacrolimus trough concentrations were available, among which 217 observations were obtained during intravenous therapy, and 215 were gathered after oral administration. The diagnoses included acute lymphatic leukemia, acute myelogenous leukemia, chronic myeloid leukemia, *β*-thalassemia major, aplastic anemia, and other types. A total of 61 patients suffered acute graft-versus-host disease (aGVHD), and nine patients had chronic graft-versus-host disease (cGVHD) after transplantation. A median of 5 (range 1–21) samples was obtained per patient. The median tacrolimus dosages were 0.024 mg kg^−1^ day^−1^ and 0.057 mg kg^−1^ day^−1^ for intravenous infusion and oral administration, respectively. The results of the baseline laboratory test are presented in Table 2. In the subpopulation data set (*n* = 24), only one patient with a wild genotype of CYP3A5*1/*1 was detected. A total of 12 of the transplanted patients showed the heterozygote genotype (*1/*3), and eleven patients showed the homozygous genotype (*3/*3) for polymorphic allele CYP3A5*1. The detailed genotypes for the subpopulation patients (*n* = 24) are shown in Supplementary Table S2.

**TABLE 1:** Patient characteristics in pediatric HSCT Recipients (*n* = 86).

Characteristics	Number (%) or Median(range)	
Sex		
Male	53	
Female	33	
Age (years)	5 (1–16)	
Weight (kg)	17.4 (6.00–50.0)	
Diagnoses		
Acute lymphatic leukemia	22(25.6%)	
*β*-thalassemia	24(27.9%)	
Acute myelogenous leukemia	17(19.8%)	
Aplastic anemia	10 (11.6%)	
Chronic myeloid leukemia	3 (3.5%)	
Juvenile myelomonocytic leukemia	2(2.3%)	
Others	8 (9.3%)	
GVHD incidence		
aGVHD	61 (70.9%)	
Grade I-II	25 (29.1%)	
Grade III-IV	36 (41.9%)	
cGVHD	9 (10.5%)	
Transplant type		
Related donor	17 (19.8%)	
Unrelated donor	69 (80.2%)	
Type of donor graft		
Bone marrow	14 (16.3%)	
Peripheral blood stem cells	49 (57.0%)	
Cord blood	35 (40.7%)	
Number of samples	578	
Intravenous dosing	320	
Oral dosing	258	
Samples per patient	5 (1–21)	
Tacrolimus dose (mg·kg^−1^·d^−1^)		
Intravenous dosing	0.024 (0.004–0.056)	
Oral dosing	0.057 (0.008–0.205)	

Results are represented as number (percentage) or median (range). GVHD.

**TABLE 2:** Clinical characteristics of patients.

Characteristics	Median (Range)
White blood cells (10^9^ L^−1^)	4.82 (0.01–25.16)
Red blood cells (10^12^ L^−1^)	3.14 (1.84–5.54)
Hemoglobin (g·L^−1^)	96.5 (54–150)
Platelets (10^9^ L^−1^)	86 (8–548)
Hematocrit (%)	0.289 (0.166–0.445)
Urea (mmol·L^−1^)	4.65 (1.6–24.6)
Serum creatinine (μmol·L^−1^)	37 (12–114)
Total protein (g·L^−1^)	60.6 (35–81.6)
Albumin (g·L^−1^)	34.65 (19–47.6)
Globulin (g·L^−1^)	24.7 (11.6–41.8)
Alanine transaminase (U·L^−1^)	53 (10–1,224)
Aspartate transaminase (U·L^−1^)	44.5 (6–644)
Total bilirubin (μmol·L^−1^)	11.3 (1.5–186.1)

### Pharmacokinetic Modeling

**FIGURE 1:**
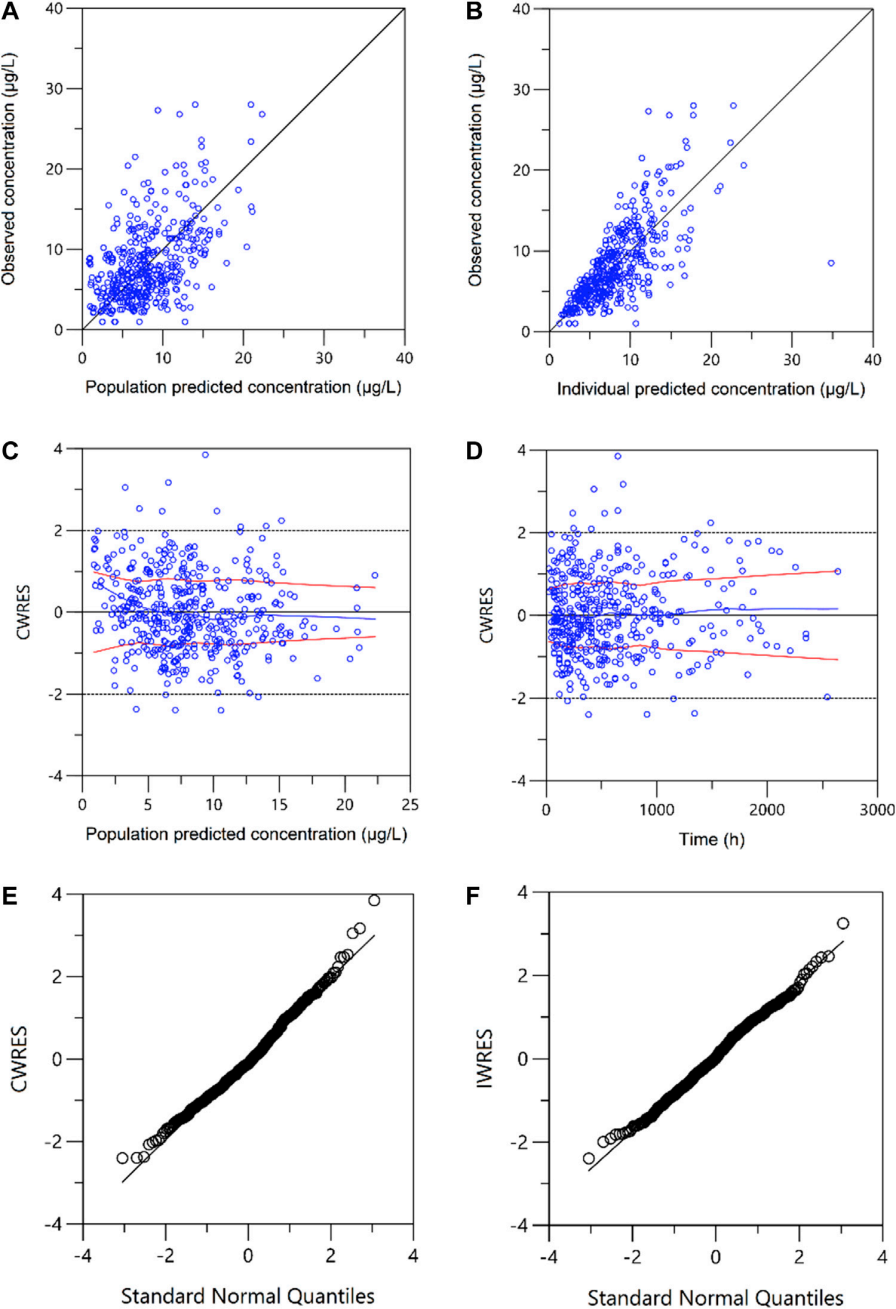
Plots of the goodness of fit. **(A)** Observed versus population-predicted concentrations (DV vs. PRED); **(B)** Observed versus individual-predicted concentrations (DV vs. IPRED); **(C)** Conditional weighted residuals versus population-predicted concentrations (CWRES vs. PRED); **(D)** Conditional weighted residuals versus time (CWRES vs. IVAR). **(E)** Conditional weighted residuals (CWRES) versus standard normal quantiles. **(F)** Individual weighted residuals (IWRES) versus standard normal quantiles.

A one-compartment with first-order absorption best described the pharmacokinetics data. A proportional residual error model was selected to explain the inter-individual variability. The visual goodness of fit plots is presented in Figure 1. The K_a_ was fixed at 4.48 according to the literature (Jusko et al., 1995; Wallin et al., 2009) since no observation was obtained at the absorption phase. During the stepwise procedures for covariate analysis, the inclusion of weight, HCT, SCr, CZ, CPFG, and PTD on CL, and HCT on V provided a significant decrease in OFV (*p* < 0.05). In the following exclusion step, SCr on CL were excluded (*p* > 0.001). Furthermore, none of the covariates investigated had a significant impact on the bioavailability. Notably, the incorporation of daily tacrolimus dosage also was tested as a covariate to explore the nonlinear clearance of the tacrolimus in the recruited patients. As a result, the inclusion of daily tacrolimus dosage as a covariate slightly improved data fitting by decreasing the OFV by 6.5 points, whereas it could not be retained in the backward elimination with an OFV increase of 5.4 points (*p* > 0.001).

Using the pharmacogenomic data set, the influence of *CYP3A5* genotypes on CL was tested in model 2. Data from 24 participants had CYP3A5 genotype information. Among those, only one out of 24 patients carried wide type homozygote, and the frequency of CYP3A5*1/*3 (*n* = 11) and CYP3A5*3/*3 (*n* = 12) was nearly 50%. In the subpopulation analysis, CYP3A5*3 presented an obvious effect on clearance; specifically, CL was 38.0% faster in patients with CYP3A5*1/*3 (*n* = 11) than in those with CYP3A5*3/*3 (*n* = 12). In addition, in patients with CYP3A5*1/*3, azoles concomitant therapy (*n* = 6) contributed to 34.3% lower CL than no treatment group (*n* = 5). No such concomitant therapy was found in patients with CYP3A5*3/*3.

The final model parameters estimates are summarized in Table 3. The final relationship describing the clearance and volume of distribution for PopPK model 1 are shown in Eqs 4 and 5 as follows:

$$\begin{array}{l} \mathrm{CL}\left(L/h\right)\;=\;2.42\times {\left(\text{WT}/17.8\right)}^{0.56}\times {\left(\mathrm{Hct}/0.296\right)}^{-0.66}\\ \;\;\;\times \exp\left(-0.40\times \left(\text{if}\;\text{with}\;\text{azoles}\;\text{comedication}\right)\right)\\ \;\;\;\times \exp\left(0.16\times \left(\mathrm{if}\;\mathrm{with}\;\mathrm{caspofungin}\;\mathrm{comedication}\right)\right)\\ \;\;\;\times \exp\left(-0.32\times \left(\text{if}\;\text{PTD}=2\right)\right), \end{array}$$ (4)

$$V\left(L\right)=79.6\times {\left(\text{Hct}/0.289\right)}^{-0.66},$$ (5)

**TABLE 3:** Population pharmacokinetic parameters of tacrolimus and bootstrap validation.

Parameters	Final Model (*n* = 86)		Bootstrap (*n* = 1,000)		Subpopulation: Pharmacogenomic Dataset (*n* = 24)	
	Estimate	*RSE* (%)	Median	95% *CI*	Estimate	*RSE* (%)
*k*_*a*_ (h^−1^)	4.48	—	4.48	—	4.48	—
*CL* (L·h^−1^)	2.42	10.84	2.43	(1.90, 3.15)	2.41	10.87
*V* (L)	79.6	16.51	80.2	(50.7, 116)	92.9	24.07
*F*	0.19	13.01	0.19	(0.15, 0.24)	0.25	20.12
*θ*_*WT, CL*_	0.56	25.65	0.59	(0.28, 0.84)	0.48	26.00
*θ*_*Hct, CL*_	-0.66	24.78	-0.63	(-1.11, -0.16)	-1.06	18.08
*θ*_*CZ, CL*_	-0.40	12.54	-0.38	(-0.60, -0.20)	-0.39	32.09
*θ*_*CPFG, CL*_	0.16	46.65	0.15	(0.01, 0.37)	0.04	37.81
*θ*_*PTD, CL*_	-0.32	38.32	-0.34	(-0.60, -0.03)	-0.33	26.75
*θ*_*Hct, V*_	-0.66	24.61	-0.64	(-1.07, -0.31)	-0.60	26.46
*θ*_CYP3A5*3, CL_	—	—	—	—	0.32	27.08
Between-subject variation						
*ω* ^*2*^_*CL*_	0.06	22.56	0.11	(0.05, 0.18)	0.02	27.97
*ω* ^*2*^_*V*_	0.66	46.27	0.72	(0.41, 1.04)	0.66	43.27
*ω* ^*2*^_*F*_	0.51	30.64	0.37	(0.09, 0.64)	0.25	51.38
Within-subject variation						
*σ*_Proportional_ (%)	37.4	3.73	36.8	(33.4, 40.4)	36.8	9.90

The final relationship describing the clearance and volume of distribution for PopPK model 2 are shown in Eqs 6 and 7 as follows:

$$\begin{array}{l} CL\left(\text{L}/\text{h}\right)=2.41\times {\left(\text{WT}/17.8\right)}^{0.48}\times {\left(\text{Hct}/0.296\right)}^{-1.06}\\ \;\;\;\;\times exp\left(-0.39\times \left(\text{if}\;\text{with azoles}\;\text{comedication}\right)\right)\\ \;\;\;\;\times exp\left(0.04\times \left(\mathrm{if}\;\mathrm{with}\;\mathrm{caspofungin}\;\mathrm{comedication}\right)\right)\\ \;\times exp\left(-0.33\times \left(\text{if PTD}=2\right)\right)\times exp\left(0.32\times \left(\text{if CYP}3\text{A}5\text{*}1\;\text{carriers}\right)\right), \end{array}$$ (6)

$$V\left(L\right)=92.9\times {\left(\text{Hct}/0.289\right)}^{-0.60},$$ (7)

where WT is the body weight, Hct is the hematocrit, and PTD = 2 is the post-transplant days≥28 days.

### Model Qualification

A non-parametric bootstrap (*n* = 1,000) was applied to validate the robustness of the final model. The successful convergence percentage was 100%. The median and 95% CI of the estimated parameters from bootstrap are shown in Table 3, which coincided well with the estimates from the final model.

**FIGURE 2:**
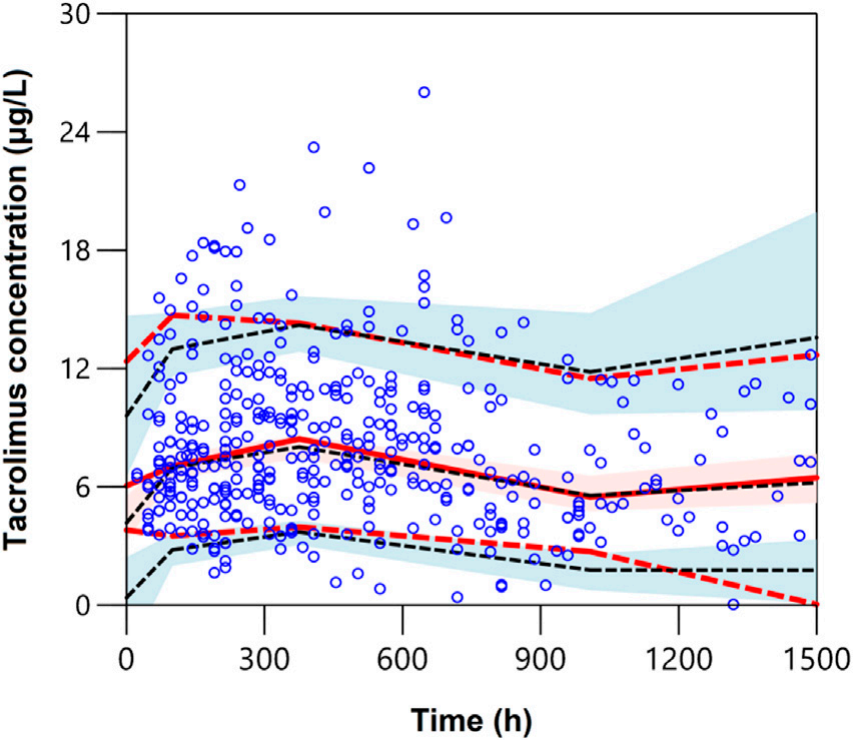
Plot of the prediction-corrected visual predictive check (*n* = 1,000). The blue dots are the measured concentrations. The dashed black lines represent the 10%, 50%, and 90% percentiles for the measured concentrations. The solid red line represents the median of simulated predictions by the final model, and the semitransparent red shaded area represents the simulation-based 90% confidence interval for the median. The red dash lines represent the 10% and 90% percentiles of simulated concentrations, and the semitransparent blue shaded areas represent the simulation-based 90% CIs for the corresponding predicted percentiles from the final model.

The pcVPCs of the final model are shown in Figure 2. The 5th, 50th, and 95th percentiles of the measured concentrations were overlaid with the 95% confidence intervals of the corresponding prediction percentiles for the final model. Less than 10% of the observations fell outside the 90% prediction intervals, indicating a robust predictive ability of the model. The pcVPC plots were cut off at 1,500 h due to limited concentrations obtained after 1,500 h. Furthermore, to assess if the estimated relationship between exposure parameters and concentrations resembles the observations, the pediatric patient population was stratified based on categorical exposure parameters (including the route of administration, age, and PTD). The stratified pc-VPC plots are presented in Supplementary Figure S1.

### Simulation

**FIGURE 3:**
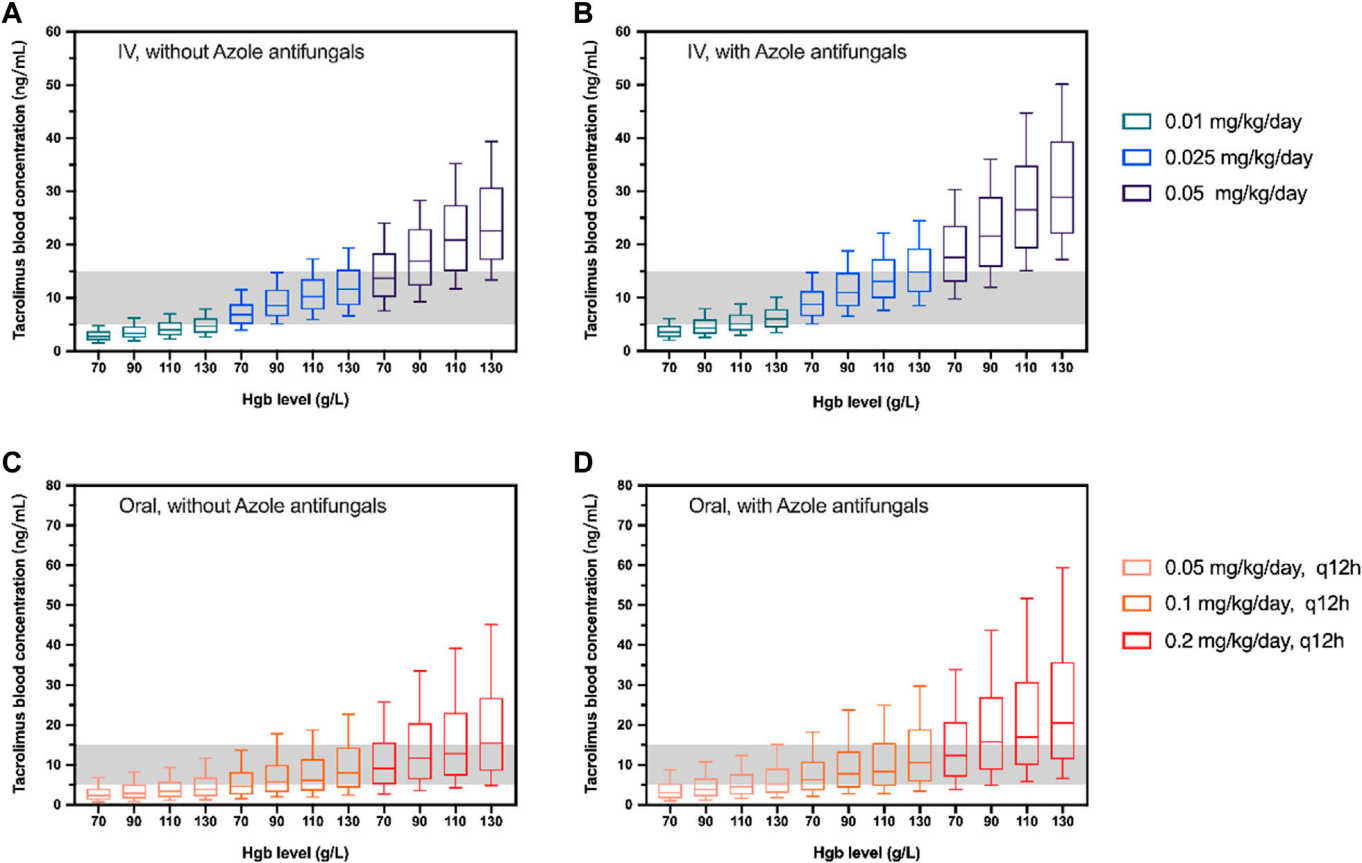
Box plots of simulated trough concentration of the typical patient (body weight 17.4 kg) with different Hgb levels following intravenous administration (IV; **(A, B)**) and oral administration (Oral; **(C, D)**). The simulated dosages for intravenous infusion were 0.01, 0.025, and 0.05 mg/kg/day and 0.05, 0.01, and 0.2 mg/kg/day(q12h) for oral administration with **(B,D)** and without azole antifungals **(A, C)**. The shadow represents tacrolimus concentrations with 5–15 ng/mL. The horizontal bars in the middle are the median values and the whiskers represent the 90% percentiles of C_0_.

The simulated dosages for intravenous infusion were 0.01, 0.025, 0.05 mg kg^−1^ day^−1^, and 0.05, 0.1, 0.2 mg kg^−1^ day^−1^ (q12h) for oral administration. Table 4 demonstrates the outcome of 1,000 simulations of the typical patient (body weight 17.4 kg, Hgb 97 g L^−1^) for different dosage regimens. The C_0_ concentrations of different Hgb levels are presented in Figure 3. The reference interval of tacrolimus concentration was 5–15 ng mL^−1^.

**TABLE 4:** Simulations (*n* = 1,000) for the typical pediatric HSCT transplant patient receiving different dosage regimens of tacrolimus.

Dosage (mg·kg·day^−1^)	*C*_0_	
	Median	10th–90th Percentile
0.01	3.6	2.1–6.4
0.025	9.2	5.2–16.3
0.05	18.3	10.6–31.2
Oral administration (q12h)		
0.05	3.0	1.0–8.4
0.1	6.1	1.9–17.8
0.2	13.2	4.1–36.1

**FIGURE 4:**
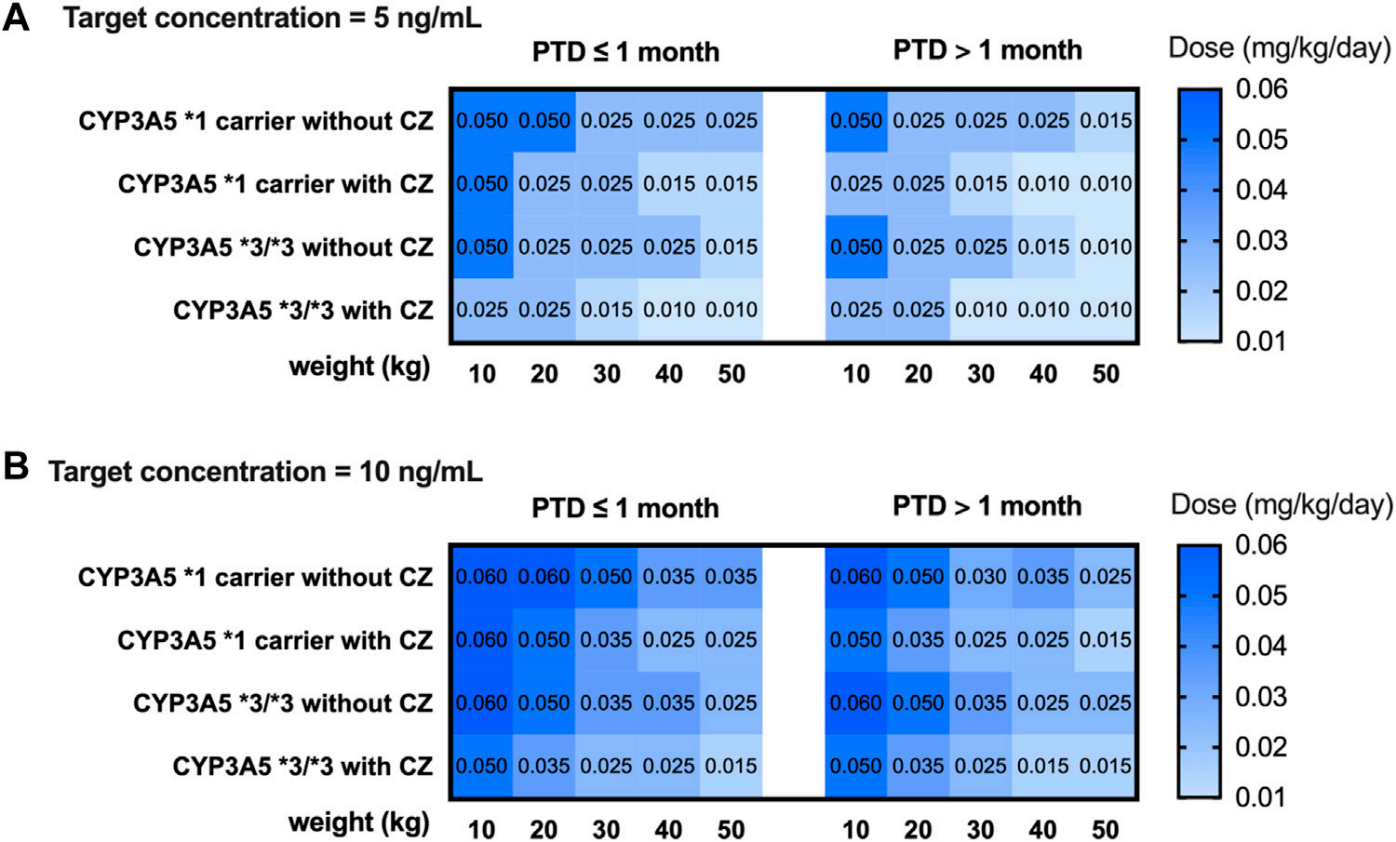
Recommended intravenous dosage of tacrolimus for HSCT children (PTD ≤ 1 month, PTD > 1 month) based on target trough concentration 5 ng/mL **(A)** and 10 ng/mL **(B)**. The simulated HSCT children are CYP3A5*1 and CYP3A5*3/*3 carriers with a weight of 10 to 50 kg.

**FIGURE 5:**
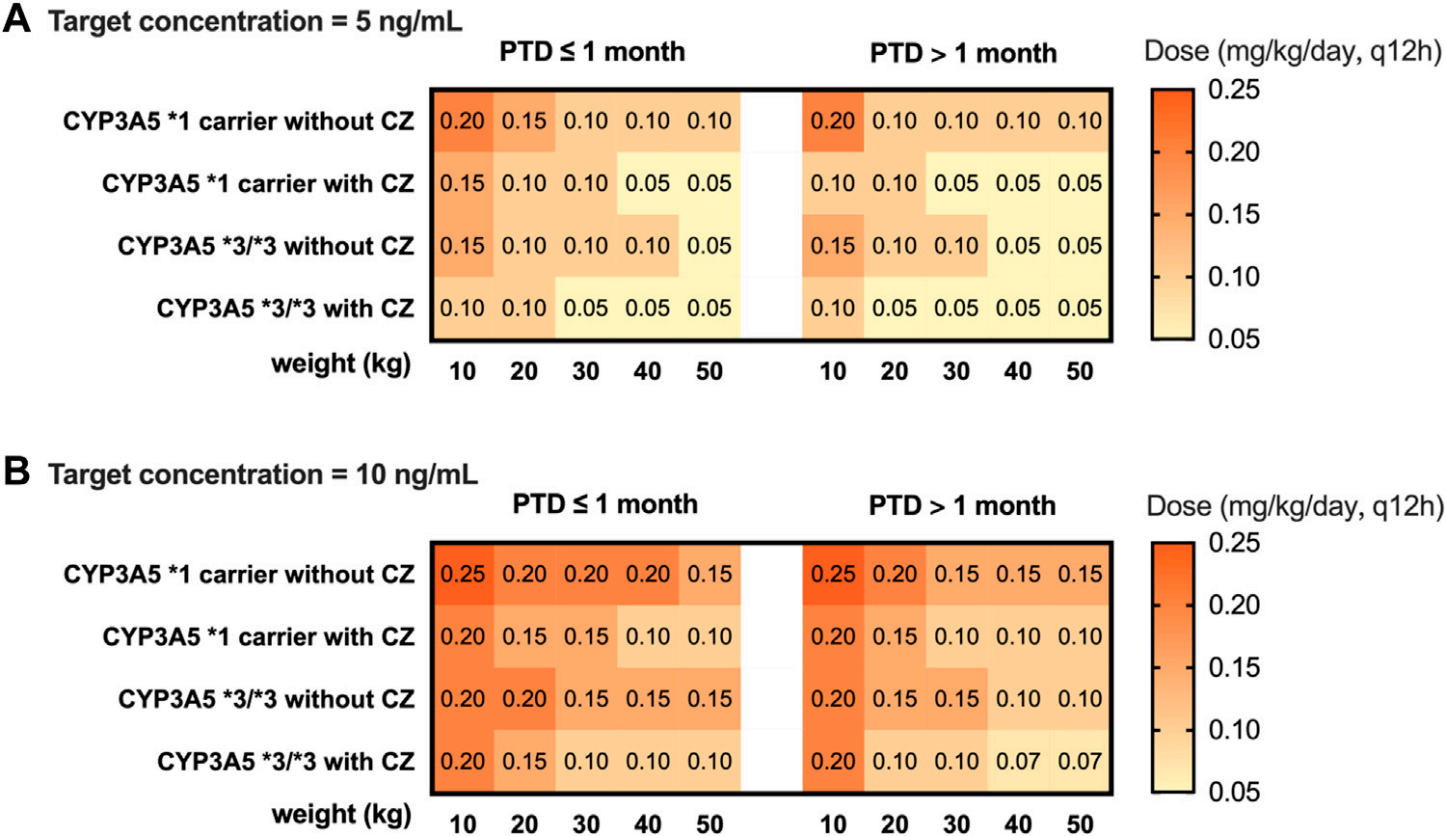
Recommended oral dosage of tacrolimus for HSCT children (PTD ≤ 1 month, PTD > 1 month) based on target trough concentration 5 ng/mL **(A)** and 10 ng/mL **(B)**. The simulated HSCT children are CYP3A5*1 and CYP3A5*3/*3 carriers with a weight of 10 to 50 kg.

A simulation based on CYP3A5 genotypes was carried out using the parameters of model 2. The C_0_ concentration of different weights, PTD, and dosages were simulated. Different dosing strategies based on covariates (PTD, CYP3A5, azoles comedication, and weight) are listed in Supplementary Tables S3, S4. Considering that the clinical target therapeutic concentration of tacrolimus varies according to the severity of GVHD, the target therapeutic concentrations were set at 5 ng mL^−1^ and 10 ng mL^−1^. The recommended intravenous and oral doses of tacrolimus for children undergoing HSCT based on the simulation are shown in Figures 4 and 5.

## Discussion

This study developed a population pharmacokinetics (PopPK) model of tacrolimus in pediatric hematopoietic stem cell transplant (HSCT) patients and devised model-guided dosage regimens. One-compartment models with and without lag time have been reported for pediatric patients of different translation types, including lung, kidney, and heart (Sam et al., 2000; Fukudo et al., 2006; Guy-Viterbo et al., 2014; Lu et al., 2015; Rower et al., 2017; Andrews et al., 2018). In this study, a one-compartment model without lag time was selected. The estimated CL in our study is 139.1 mL h^−1^ kg^−1^, which is in good agreement with that in bone marrow transplantation (BMT) children (108.1 ml h^−1^ kg^−1^, range 79.7–142.0 ml h^−1^ kg^−1^) (Mehta et al., 1999). It is reported that tacrolimus clearance is age-dependent within the pediatric population and the mean clearance was 159 ± 82, 109 ± 53, and 104 ± 68 ml h^−1^ kg^−1^ for patients <6 years, 6–12 years, and >12 years, respectively (Przepiorka et al., 2000). The total body clearance was 71 ± 34 ml h^−1^ kg^−1^ in adult BMT patients (Fay et al., 1996). However, age-dependent clearance was not observed in our study. The estimated volume of distribution was 70 L, and the bioavailability was 21%. The between-subject variability (BSV) of CL, V, and F was estimated to be 45%, 98%, and 72%, respectively, which is similar to other previously reported population analyses (Wallin et al., 2009; Xue et al., 2009). Tacrolimus showed a moderate inter-subject variability in clearance, while the volume of distribution and bioavailability were associated with higher variability. To date, inter-individual variations in the expression of CYP3A5 are thought to be the main factor responsible for inter-individual variability; indeed, genetic polymorphism is considered the cause of high variability of tacrolimus blood concentrations for a given dose. However, no related genetic polymorphism was found to have significant influences on the bioavailability, which, to some extent, can be contributed by a relatively small sample size (*n* = 24) in patients with CYP3A5 genotypes. Otherwise, previous studies have demonstrated an inverse relationship between bioavailability and inter-individual variability, so when the bioavailability is low, a large inter-individual variability will be expected (Hellriegel et al., 1996). To further evaluate the estimated relationship between stratified parameters and observations, the pc-VPCs were obtained after dividing into two different subgroups for each exposure parameter. However, inflations were observed in the upper prediction intervals when the stratified parameters were oral administration, PTD>1 month and age>6 years (Supplementary Figure S1). Obviously, according to scatters in the pc-VPC plots, the blood samples collected during the period of 1,000–1,500 h were insufficient though the *x*-axis of pcVPC plots extended to 1,500 h. It would have been more information if pc-VPCs allowed the direct comparison between model-simulated and observed concentrations under the different subgroups, and further development is needed in a large population sample.

In this present study, our results displayed a negative relationship between hematocrit levels and tacrolimus clearance after hematopoietic stem cell transplantation, which is consistent with findings from several studies (Guy-Viterbo et al., 2013; Andrews et al., 2018). Other retrospective cohorts also have shown that hematocrit (or hemoglobin) is associated with the volume of distribution in a negative correlation in HSCT patients (Xue et al., 2009; Yoshida et al., 2021). This is because of the strong binding of tacrolimus to erythrocytes, facilitated by the drug’s lipophilicity and the presence of immunophilins in red blood cells. Thus, a low hematocrit results in a reduction of tacrolimus concentrations in blood (Staatz and Tett, 2004). It is important to note that, in the early phase after HSCT, red blood cell (RBC) transfusions are necessary due to RBC counts are dramatically decreased. Therefore, RBC counts in HSCT patients vary widely during the treatment period. Hemoglobin could be a great substitute for hematocrit, which could improve the estimation of whole blood levels with Hgb monitoring (Yoshida et al., 2021). In our study, a strong relationship was found between Hct and Hgb with a calculation formula of HCT = 0.0029*Hgb+ 0.0038 (*r* ^2^ = 0.9527, Supplementary Figure S2). Consequently, the Hgb level was used as a reliable index of hematocrit and C_0_ under different Hgb levels (70, 90, 110, and 130 g L^−1^) was simulated to illustrate the influence of Hgb level on the tacrolimus dosage choosing on HSCT patients (Figure 3).

Different concomitant medications in immunosuppressant regimens after HSCT transplantation could have an impact on the pharmacokinetics of tacrolimus by affecting the action of CYP3A4 or P-gp, such as voriconazole, itraconazole, and posaconazole (Cervelli and Russ, 2003; Rower et al., 2017; Huang et al., 2020). In our investigation, concomitant voriconazole use was found to be a major covariate that explains interindividual variability in tacrolimus CL, which is consistent with other PopPK studies. Voriconazole is a CYP3A inhibitor, and, thus, its treatment combination with tacrolimus may lead to a reduction in the biotransformation. The concomitant medications of these drugs reduce the CL and increase the blood concentration. It is important to note that there were 41 (50% overall) children who administrated concomitant azole use but only 6 (25%) children in the subpopulation cohort, and all received it *via* IV administration. In the subgroup, children who received concomitant medications across medical centers could lead to variations in the drug’s disposal under different CYP3A5 genotypes. It is to be noted that participants available for model building (subpopulation) appear to be relatively small, and the drug–drug or gene–drug interactions are required to be explained in future studies after enrolling a large cohort. In addition, we did not have information about the azole dose received and the related severity of the illness.

Genetic variation of CYP3A5 enzyme can explain up to 40%–50% of tacrolimus clearance variation (Tang et al., 2016). CYP3A5*3 has been implicated as a significant predictor of tacrolimus pharmacokinetics (Zuo et al., 2013; Zhang et al., 2015; Andreu et al., 2017; Sanghavi et al., 2017). Various retrospective cohort studies suggest that the clearance of CYP3A5*1/*1 carriers is higher than that of CYP3A5*3/*3 homozygotes (1.15–2.5-fold) (Campagne et al., 2019). However, to our knowledge, no such PopPK study related to the genotype of CYP3A5 exists in HSCT populations. Our observation demonstrated that clearance of tacrolimus for pediatric HSCT patients was 1.38-fold higher in CYP3A5*1 carriers than in non-carriers (CYP3A5*3/*3), which was slightly lower than the reported values. In this population, the oral bioavailability of tacrolimus is generally poor (median 19%). Tacrolimus is metabolized by cytochrome P450 3A4 and 3A5 enzymes in the gut wall and liver, which supports the fact that the bioavailability of tacrolimus is reduced in CYP3A5*1 carriers. Moreover, as biases from the analytical method and the small sample size of CYP3A5*1/*1 genotype in this study, the influence of CYP3A5 expression type on CL may be underestimated. Currently, the detection of CYP3A5 genotype has not yet been the standard clinical practice when prescribing for HSCT patients. Recent studies support the use of CYP3A5 genotyping in patients undergoing allogeneic HCT (Zhu et al., 2020; Yoshikawa et al., 2021), and our study further helps to guide tacrolimus dosing based on the PopPK model. Postoperative days have been identified as a major surrogate for many time-dependent variables (Wallemacq et al., 1998). In the present study, a gradual improvement in metabolic function with postoperative days increased the CL after hematopoietic stem cell transplantation. This may be attributed to the progressive recovery of CYP3A enzyme activity in the liver and intestine and the tapering of corticosteroid doses with increasing time after transplantation, especially during the first few weeks after transplantation. Other factors and possible mechanisms related to the influence of postoperative days on tacrolimus pharmacokinetics are still being investigated.

The initial oral dosages for kidney, liver, and heart transplant patients (adult and pediatric) have been prescribed in PROGRAF^®^ package inserts, ranging from 0.15 mg kg^−1^ day^−1^ to 0.3 mg kg^−1^ day^−1^ (Astellas, 2019). Yet there remains uncertainty on the appropriate dosage for HSCT patients. Tacrolimus is generally given by IV infusion at a dose of 0.03 mg kg^−1^ day^−1^ initially and covert to oral administration with a ratio of 1:3 to 1:5 thereafter (Brunet et al., 2019). In most clinical trials/retrospective studies, the target C_0_ concentration of tacrolimus for GVHD treatment is 10–20 ng mL^−1^ (Mori et al., 2012; Yano et al., 2015; Kanda et al., 2016). A study on children with HSCT found that during the first 4 weeks of continuous infusion of tacrolimus, an average concentration of ≤7 ng mL^−1^ would increase the risk of aGVHD and reduce the survival rate after transplantation (Watanabe et al., 2010). Another retrospective study on children showed that the mean C_0_ concentration <10 ng mL^−1^ at the third week was associated with an increased incidence of aGVHD (Offer et al., 2015). Many other studies have shown that tacrolimus concentration >20 ng mL^−1^ is related to nephrotoxicity and neurotoxicity after HSCT transplantation (Wingard et al., 1998; Przepiorka et al., 1999). The median dosage of tacrolimus from our collected data is 0.024 mg kg^−1^ day^−1^ and 0.057 mg kg^−1^ day^−1^, for IV and oral, respectively. Yet approximately 40% of concentrations are under 5 ng mL^−1^ (Supplementary Figure S3), which reveals that the current dosing might be insufficient. According to our simulation, the tacrolimus C_0_ falls within the therapeutic window of 5–15 ng mL^−1^ when given 0.025 mg kg^−1^ day^−1^ by IV infusion without azole antifungals, whilst the proper oral dosage is 0.1 mg kg^−1^ day^−1^, q12h (Figure 3). Also, the results of the simulated C_0_ indicate that the proposed 0.3 mg kg^−1^ day^−1^ for pediatric kidney or heart transplant patients might be too high for those who received HSCT.

Our study developed a PopPK model to characterize the pharmacokinetics of tacrolimus and to derive a model-based dosage regimen in pediatric HSCT patients; however, in this clinical setting, the absorption phase and terminal elimination of tacrolimus could not be estimated accurately because only trough concentrations were collected during the study. Another limitation of our study was that the PopPK model of tacrolimus was internally validated only in our study. Ultimately, the model should be validated externally in another cohort of pediatric HSCT patients, and a prospective study to verify the recommended dosage regimens in this study is needed in the future. Nevertheless, the PopPK model exhibited a shrinkage value related to interindividual variability on clearance of 10.84%, illustrating the good quality of its model-building procedure, which could be associated with an appropriate number of observations per patient used for data analysis in this study. As reported that a value of shrinkage ≤20% clarifies that the relationships between clearance and the covariates are reliable (Mould and Upton, 2013). Despite all the abovementioned limitations, the study can provide a theoretical basis for individualized tacrolimus therapy and serve as a good reference for pediatric HSCT patients.

## Conclusion

A population PK model was developed and validated to describe the tacrolimus pharmacokinetics in pediatric HSCT recipients. Bodyweight, hematocrit, CYP3A5 genotypes, post-transplantation day, and the combination of azole antifungal agents had a significant influence on the clearance of tacrolimus. Intravenous infusion of 0.025 mg kg^−1^ day^−1^ and oral 0.1 mg kg^−1^ day^−1^ (q12h) is relatively appropriate. Subsequently, a more targeted visual initial dose recommendation table was developed, which may be helpful for the tacrolimus initial dose determination for HSCT children and improve routine therapeutic drug monitoring.

## Data Availability

The raw data supporting the conclusions of this article will be made available by the authors, without undue reservation.
