# Supplementary material for: Population Pharmacokinetics and Initial Dosage Optimization of Tacrolimus in Pediatric Hematopoietic Stem Cell Transplant Patients
Source: Front Pharmacol. 2022 Jul 6;13:891648. doi: 10.3389/fphar.2022.891648 (PMC9298550; doi:10.3389/fphar.2022.891648)
Supplement: Supplementary file 1 [file Table1.docx]

**Supplemental Table 1. Primer sequences**

| ***CYP3A5 rs776746*** | | **Primer sequence** |
| --- | --- | --- |
| Forward primer sequence | ACGTTGGATGGTAATGTGGTCCAAACAGGG | |
| Reverse primer sequence | ACGTTGGATGATGTACCACCCAGCTTAACG | |
| Extend primer sequence | GGTCCAAACAGGGAAGAGATA | |
