# Supplementary figures and images for: Population Pharmacokinetics and Initial Dosage Optimization of Tacrolimus in Pediatric Hematopoietic Stem Cell Transplant Patients
Source: Front Pharmacol. 2022 Jul 6;13:891648. doi: 10.3389/fphar.2022.891648 (PMC9298550; doi:10.3389/fphar.2022.891648)

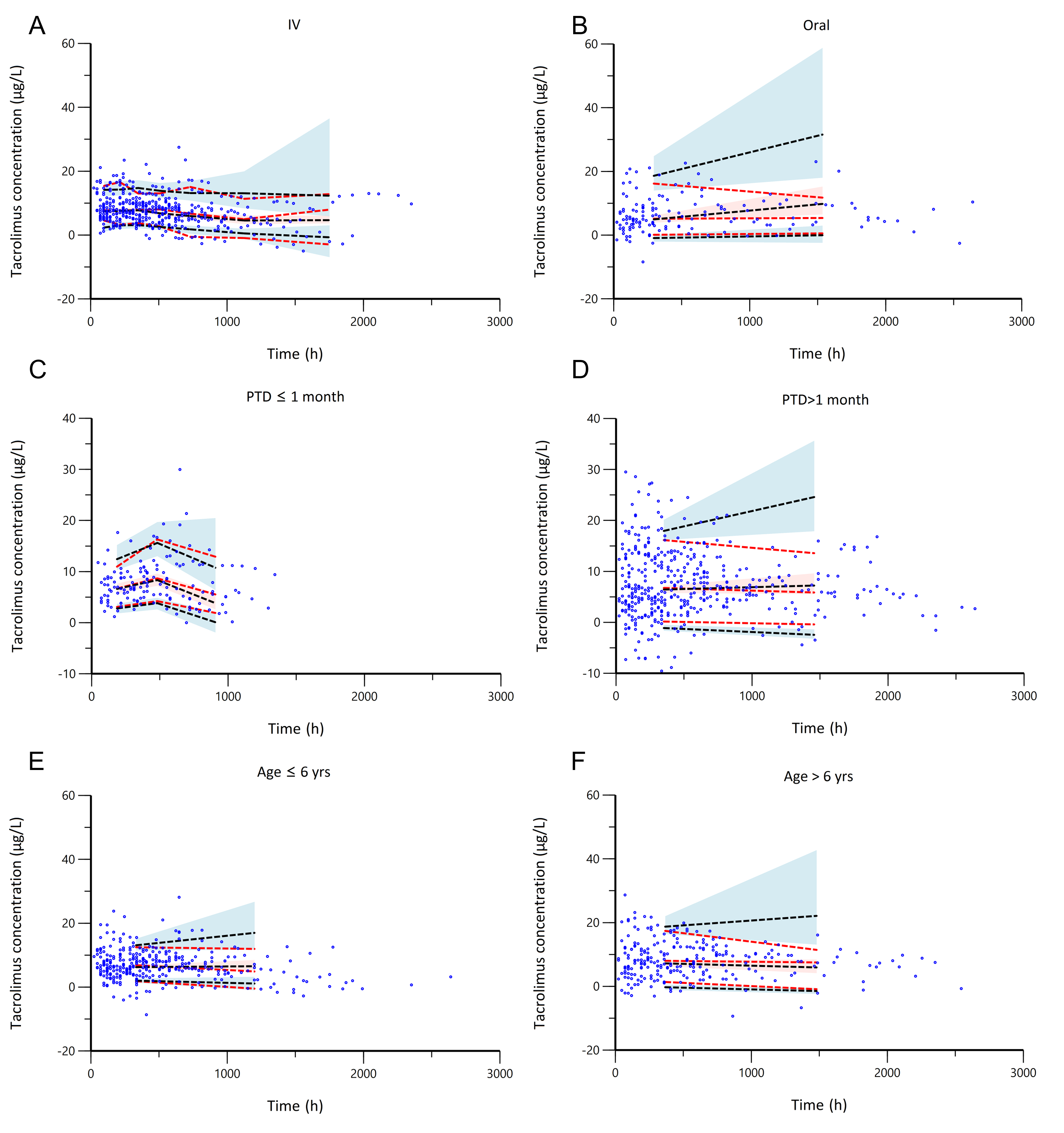

Supplement: Supplementary file 2 [file Image3.TIF]

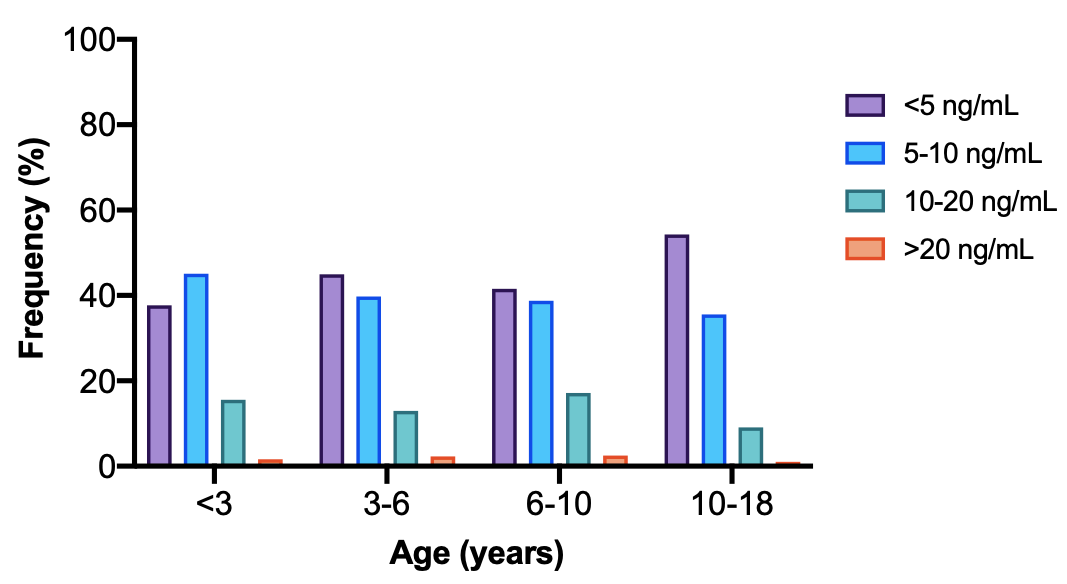

Supplement: Supplementary file 3 [file Image2.TIF]

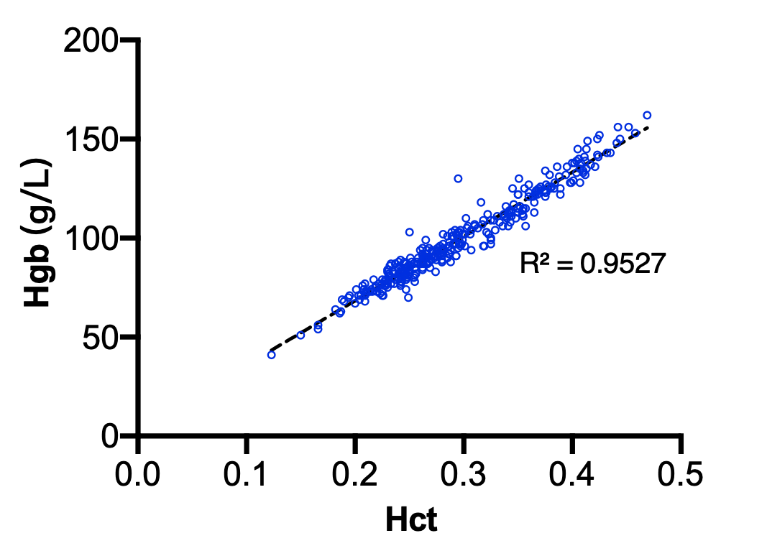

Supplement: Supplementary file 4 [file Image1.TIF]
