# Supplementary material for: Population Pharmacokinetics and Initial Dosage Optimization of Tacrolimus in Pediatric Hematopoietic Stem Cell Transplant Patients
Source: Front Pharmacol. 2022 Jul 6;13:891648. doi: 10.3389/fphar.2022.891648 (PMC9298550; doi:10.3389/fphar.2022.891648)
Supplement: Supplementary file 5 [file Table2.docx]

**Supplemental Table 2. The genotypes of each pediatric HSCT patients in model 2(n=24).**

| **Patient ID** | ***SNP rs776746*** | |
| --- | --- | --- |
| Patient 1 | | CT |
| Patient 2 | | CC |
| Patient 3 | | CC |
| Patient 4 | | CT |
| Patient 5 | | CT |
| Patient 6 | | TT |
| Patient 7 | | CT |
| Patient 8 | | CC |
| Patient 9 | | CC |
| Patient 10 | | CC |
| Patient 11 | | CC |
| Patient 12 | | CC |
| Patient 13 | | CT |
| Patient 14 | | CC |
| Patient 15 | | CT |
| Patient 16 | | CT |
| Patient 17 | | CC |
| Patient 18 | | CC |
| Patient 19 | | CT |
| Patient 20 | | CT |
| Patient 21 | | CC |
| Patient 22 | | CT |
| Patient 23 | | CT |
| Patient 24 | | CC |
