# Supplementary material for: Population Pharmacokinetics and Initial Dosage Optimization of Tacrolimus in Pediatric Hematopoietic Stem Cell Transplant Patients
Source: Front Pharmacol. 2022 Jul 6;13:891648. doi: 10.3389/fphar.2022.891648 (PMC9298550; doi:10.3389/fphar.2022.891648)
Supplement: Supplementary file 6 [file Table3.docx]

**Supplemental Table 3. Recommended intravenous dosage of tacrolimus for HSCT children based on target trough concentration**

| Post-transplantation days | *CYP3A5* genotypes | Azoles antifungal agents | Weight (kg) | Target concentration | |
| --- | --- | --- | --- | --- | --- |
|  |  |  |  | 5 ng/mL | 10 ng/mL |
|  |  |  |  | Dosage (mg/kg/day) | Dosage (mg/kg/day) |
| ≤ 1 month | *1/*1 or *1/*3 | No | 10 | 0.050 | 0.060 |
|  |  |  | 20 | 0.050 | 0.060 |
|  |  |  | 30 | 0.025 | 0.050 |
|  |  |  | 40 | 0.025 | 0.035 |
|  |  |  | 50 | 0.025 | 0.035 |
|  | *1/*1 or *1/*3 | Yes | 10 | 0.050 | 0.060 |
|  |  |  | 20 | 0.025 | 0.050 |
|  |  |  | 30 | 0.025 | 0.035 |
|  |  |  | 40 | 0.015 | 0.025 |
|  |  |  | 50 | 0.015 | 0.025 |
|  | *3/*3 | No | 10 | 0.050 | 0.060 |
|  |  |  | 20 | 0.025 | 0.050 |
|  |  |  | 30 | 0.025 | 0.035 |
|  |  |  | 40 | 0.025 | 0.035 |
|  |  |  | 50 | 0.015 | 0.025 |
|  | *3/*3 | Yes | 10 | 0.025 | 0.050 |
|  |  |  | 20 | 0.025 | 0.035 |
|  |  |  | 30 | 0.015 | 0.025 |
|  |  |  | 40 | 0.010 | 0.025 |
|  |  |  | 50 | 0.010 | 0.015 |
| > 1 month | *1/*1 or *1/*3 | No | 10 | 0.050 | 0.060 |
|  |  |  | 20 | 0.025 | 0.050 |
|  |  |  | 30 | 0.025 | 0.030 |
|  |  |  | 40 | 0.025 | 0.035 |
|  |  |  | 50 | 0.015 | 0.025 |
|  | *1/*1 or *1/*3 | Yes | 10 | 0.025 | 0.050 |
|  |  |  | 20 | 0.025 | 0.035 |
|  |  |  | 30 | 0.015 | 0.025 |
|  |  |  | 40 | 0.010 | 0.025 |
|  |  |  | 50 | 0.010 | 0.015 |
|  | *3/*3 | No | 10 | 0.050 | 0.060 |
|  |  |  | 20 | 0.025 | 0.050 |
|  |  |  | 30 | 0.025 | 0.035 |
|  |  |  | 40 | 0.015 | 0.025 |
|  |  |  | 50 | 0.010 | 0.025 |
|  | *3/*3 | Yes | 10 | 0.025 | 0.050 |
|  |  |  | 20 | 0.025 | 0.035 |
|  |  |  | 30 | 0.010 | 0.025 |
|  |  |  | 40 | 0.010 | 0.015 |
|  |  |  | 50 | 0.010 | 0.015 |
